# Genome‐wide identification and analysis of heterotic loci in three maize hybrids

**DOI:** 10.1111/pbi.13186

**Published:** 2019-06-27

**Authors:** Hongjun Liu, Qin Wang, Mengjiao Chen, Yahui Ding, Xuerong Yang, Jie Liu, Xiaohan Li, Congcong Zhou, Qilin Tian, Yiqi Lu, Danlin Fan, Junpeng Shi, Lin Zhang, Congbin Kang, Mingfei Sun, Fangyuan Li, Yujian Wu, Yongzhong Zhang, Baoshen Liu, Xiang Yu Zhao, Qi Feng, Jinliang Yang, Bin Han, Jinsheng Lai, Xian Sheng Zhang, Xuehui Huang

**Affiliations:** ^1^ State Key Laboratory of Crop Biology College of Life Sciences Shandong Agricultural University Tai'an China; ^2^ Shanghai Key Laboratory of Plant Molecular Sciences College of Life Sciences Shanghai Normal University Shanghai China; ^3^ National Center for Gene Research CAS Center for Excellence of Molecular Plant Sciences Institute of Plant Physiology and Ecology Shanghai Institutes for Biological Sciences Chinese Academy of Sciences Shanghai China; ^4^ State Key Laboratory of Agrobiotechnology and National Maize Improvement Center Department of Plant Genetics and Breeding China Agricultural University Beijing China; ^5^ College of Agriculture Northeast Agricultural University Harbin China; ^6^ State Key Laboratory of Crop Biology College of Agronomy Shandong Agricultural University Tai'an China; ^7^ Department of Agronomy and Horticulture University of Nebraska‐Lincoln Lincoln NE USA

**Keywords:** heterosis, maize, genomics, quantitative trait loci, molecular breeding

## Abstract

Heterosis, or hybrid vigour, is a predominant phenomenon in plant genetics, serving as the basis of crop hybrid breeding, but the causative loci and genes underlying heterosis remain unclear in many crops. Here, we present a large‐scale genetic analysis using 5360 offsprings from three elite maize hybrids, which identifies 628 loci underlying 19 yield‐related traits with relatively high mapping resolutions. Heterotic pattern investigations of the 628 loci show that numerous loci, mostly with complete–incomplete dominance (the major one) or overdominance effects (the secondary one) for heterozygous genotypes and nearly equal proportion of advantageous alleles from both parental lines, are the major causes of strong heterosis in these hybrids. Follow‐up studies for 17 heterotic loci in an independent experiment using 2225 F_2_ individuals suggest most heterotic effects are roughly stable between environments with a small variation. Candidate gene analysis for one major heterotic locus (*ub3*) in maize implies that there may exist some common genes contributing to crop heterosis. These results provide a community resource for genetics studies in maize and new implications for heterosis in plants.

## Introduction

Heterosis, or known as hybrid vigour, refers to the phenomenon where the F_1_ hybrid has greater phenotypic performance than both inbred parents (Bruce, 1910; East, 1936; Jones, 1917; Shull, 1908). Heterosis forms the foundation of modern breeding (Birchler *et al*., 2016). As the widespread of the phenomenon, heterosis has been discovered and used in production for many crop species, such as maize (*Zea mays*), rice (*Oryza sativa*) and canola (*Brassica napus*). During recent decades, the development of a number of elite hybrids in these crops had greatly contributed to the food security through increasing crop yield per unit area across the world (Schnable and Springer, 2013). To further exploit heterosis potentials in hybrids by a genomics‐assisted breeding (GAB), the understanding of heterosis mechanisms, coupled with the discovery of the major genetic loci involved in yield heterosis performances in the major crops, will be of great interests.

The molecular basis of heterosis has been explored from different perspectives for many decades (Garcia *et al*., 2008; Hua *et al*., 2002; Ko *et al*., 2016; Kusmec *et al*., 2017; Lu *et al*., 2003; Luo *et al*., 2011; Ma *et al*., 2018; Tang *et al*., 2010; Wang *et al*., 2016, 2019). Several major models for heterosis have been proposed, including dominance complementation and single‐locus overdominance. In the model for dominance complementation, beneficial and dominant alleles from one parental line compensate for deleterious and recessive alleles from the other. For the case of single‐locus overdominance, the heterozygous genotype has a better performance than both homozygous genotypes (Hollick and Chandler, 1998). Recently, in a number of plant species (e.g. *Arabidopsis*, rice, maize, sorghum and tomato), genetic mapping has been used to identify the detailed gene loci contributing to hybrid performances and investigate their mechanisms (Swanson‐Wagner *et al*., 2006; Riedelsheimer *et al*., 2012; Zhou *et al*., 2012; Yao *et al*., 2013; Dapp *et al*., 2015; Birchler *et al*., 2016; Li *et al*., 2016; Yang *et al*., 2017a,2017b; Liu et al., 2019). For example, in tomato and rice, the orthologue gene of *Arabidopsis* flowering locus *SFT* for tomato (Krieger *et al*., 2010) and *Hd3a* for rice (Huang *et al*., 2016), respectively, has been found to show single‐gene overdominance. In another case, repulsion linkage between two quantitative trait loci (QTLs) underlying plant height (*qHT7.1* and *Dw3*) with dominance effects caused the occurrence of heterosis in sorghum (Li *et al*., 2015).

**Figure 1:**
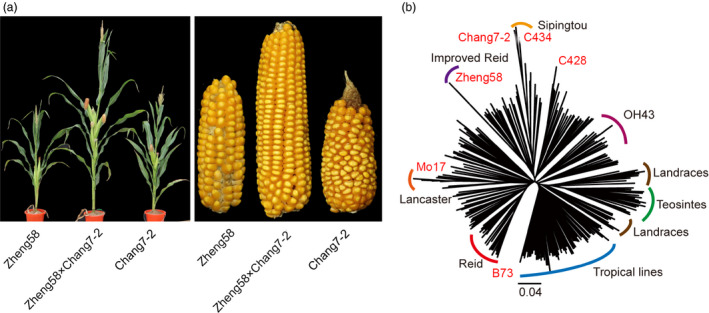
Heterosis advantage of maize hybrids and whole‐genome variation of their parental lines. (a) Phenotype of the maize hybrid Zhengdan958 and its parental inbred lines. The plant height and ears of inbred lines Zheng58 (left), Chang7‐2 (right) and the F_1_ (middle) are shown. (b) The phylogenetic positions of six parental lines (Zheng58, Chang7‐2, B73, Mo17, C428 and C434) in the neighbour‐joining tree of 287 maize inbred lines, in which the known information on the clades of maize germplasm is indicated.

Maize has been a model plant species for genetic studies of heterosis over a century, largely owing to its outcrossing habits, the availability of diverse germplasm and the importance of maize yield in global agricultural production (Riedelsheimer *et al*., 2012). Nowadays, the majority of maize varieties grown throughout the world are hybrids crossed between two inbred lines with contrasting pedigree relationships. In the United States, the maize germplasm was classified into two major heterotic groups – Stiff Stalk (SS) and Non‐Stiff Stalk (NSS). Inbred parents selected from these two heterotic groups contribute most of the genetic gains in hybrid production. Normally, these maize hybrids are usually much taller and have larger ears than the inbred parents (Figure 1a). Here, using 5360 offsprings from three elite hybrids in maize, we performed a large‐scale genetic mapping and heterosis analysis, aiming to identify important loci contributing to heterosis advantage in elite maize hybrids and provide a useful data resource for heterosis studies in plants.

## Results

### Population construction and genome sequencing

Three representative hybrid crosses in maize, Zheng58 × Chang7‐2, B73 × Mo17 and C428 × C434, were selected in this study. The cultivar Zhengdan958, which is the F_1_ plant from the cross between two inbred parental lines Chang7‐2 and Zheng58 (Figure 1a), displays a high yield performance and is the most widely grown hybrid in China (account for 13% of the maize planting areas in China since 2004). The hybrid cross between Mo17 and B73 is a classical combination in heterosis studies, especially in the United States. Another hybrid Siyu‐2 from the C428 × C434 cross is a newly developed maize cultivar in China, and the parental line C434 is derived from Chang7‐2 in breeding. The six inbred parental lines were sequenced using Illumina platform with more than 30 × coverage for each. The resequencing reads of six parental lines were combined with published data of 281 diverse maize lines (including teosintes, landraces and modern cultivars from different periods of breeding history in the United States and China; Chia *et al*., 2012; Jiao *et al*., 2012; Bukowski *et al*., 2018) to construct a neighbour‐joining tree using 2.67 million whole‐genome SNPs (minor allele frequency > 0.05). As expected, teosintes were grouped into a single clade which was surrounded by the landraces and tropical lines. All the six parental lines were identified as temperate lines from five genetically distinct clades, including those of well‐known heterotic groups of Reid (B73), Lancaster (Mo17), Improved Reid (Zheng58) and Sipingtou (Chang7‐2 and C434, Figure 1b). We further used the sequencing data to identify high‐quality SNPs for each hybrid cross. There were totally 6 807 279 SNPs (between Chang7‐2 and Zheng58), 5 285 206 SNPs (between Mo17 and B73) and 4 094 055 SNPs (between C434 and C428) across the maize genome (AGPv4). These SNPs between parents covered the majority of genomic regions, thus facilitating follow‐up genotyping in the F_2_ populations (Figure S1a‐c). Interestingly, there were two very large *identical by state* (IBS) segments on chromosome 2 (located within the 53–183 Mb interval) and chromosome 7 (located within the 8–128 Mb interval) between C434 and C428 covering over half of the chromosomes (Figure S1c), indicating that the parental lines C428 and C434 had a shared common ancestor in the recent breeding.

**Figure 2:**
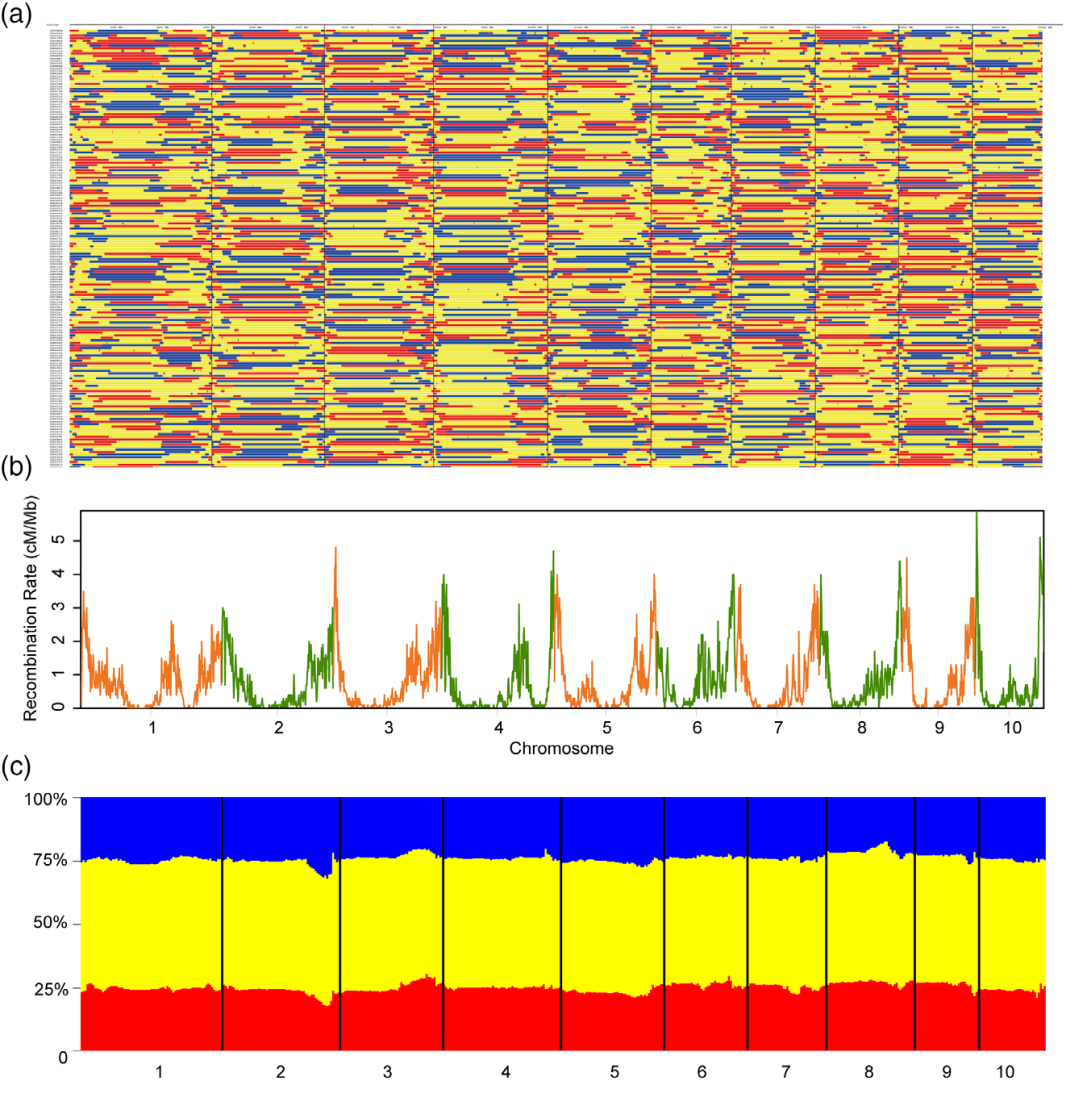
High‐resolution genotyping of F_2_ lines by whole‐genome resequencing and the distribution of recombination rates in the hybrid Zhengdan958. (a) The recombination bin map of the F_2_ population (*n *=* *2567) from a cross between Zheng58 and Chang7‐2, in which the horizontal axis indicates the genomic regions and the vertical axis indicates the F_2_ lines. Zheng58/Zheng58 homozygous type is shown in red, Zheng58/Chang7‐2 heterozygous type is shown in yellow, and Chang7‐2/Chang7‐2 homozygous type is shown in blue. (b) Plots of the recombination rates of the Zhengdan958 F_2_ population throughout the maize chromosomes. (c) Statistic of separation ratio of the three genotypes. Zheng58/Zheng58 homozygous type is shown in red, Zheng58/Chang7‐2 heterozygous type is shown in yellow, and Chang7‐2/Chang7‐2 homozygous type is shown in blue.

We then generated three sets of large F_2_ populations from the F_1_ hybrid combinations, with 2567 F_2_ individuals, 1505 F_2_ individuals and 1288 F_2_ individuals for Zheng58 (Female) × Chang7‐2 (Male), B73 (Female) × Mo17 (Male) and C428 (Male) × C434 (Female), respectively. The genomes of all the 5360 lines were sequenced individually using Illumina platform, each with ~0.2× coverage, resulting in a total of ~2 Tb sequence data. The raw reads of each F_2_ individuals were aligned to the maize reference genome for genotype calling. Using the software SEG‐Map, high‐density genetic maps were generated subsequently for each population (Figure 2; Huang *et al*., 2009). In the F_2_ population of Zheng58 × Chang7‐2, the total length of the genetic map is 1588.8 cM. For each F_2_ individuals in Zheng58 × Chang7‐2, there were generally 2–4 recombination events on each chromosome (Figure 2a). We observed that the recombination varied extremely along chromosomes – all chromosomes had very low recombination rates around peri‐centromeric regions, and high recombination rates around peri‐telomere regions (Figure 2b, the plots for Zhengdan958 population), which is consistent with the recombination pattern evaluated from maize haplotype map (Bukowski *et al*., 2018; Chia *et al*., 2012; Rodgers‐Melnick *et al*., 2015). The exceptions occurred on chromosomes 1 and 4 – we found that besides the peri‐telomere region, there was another region with extremely low recombination rates (located within the 224–245 Mb interval of chromosome 1 and the 201–228 Mb interval of chromosome 4), probably suggesting the existence of some other factors to restrict the recombination (e.g. large genomic inversions between parental lines).

**Figure 3:**
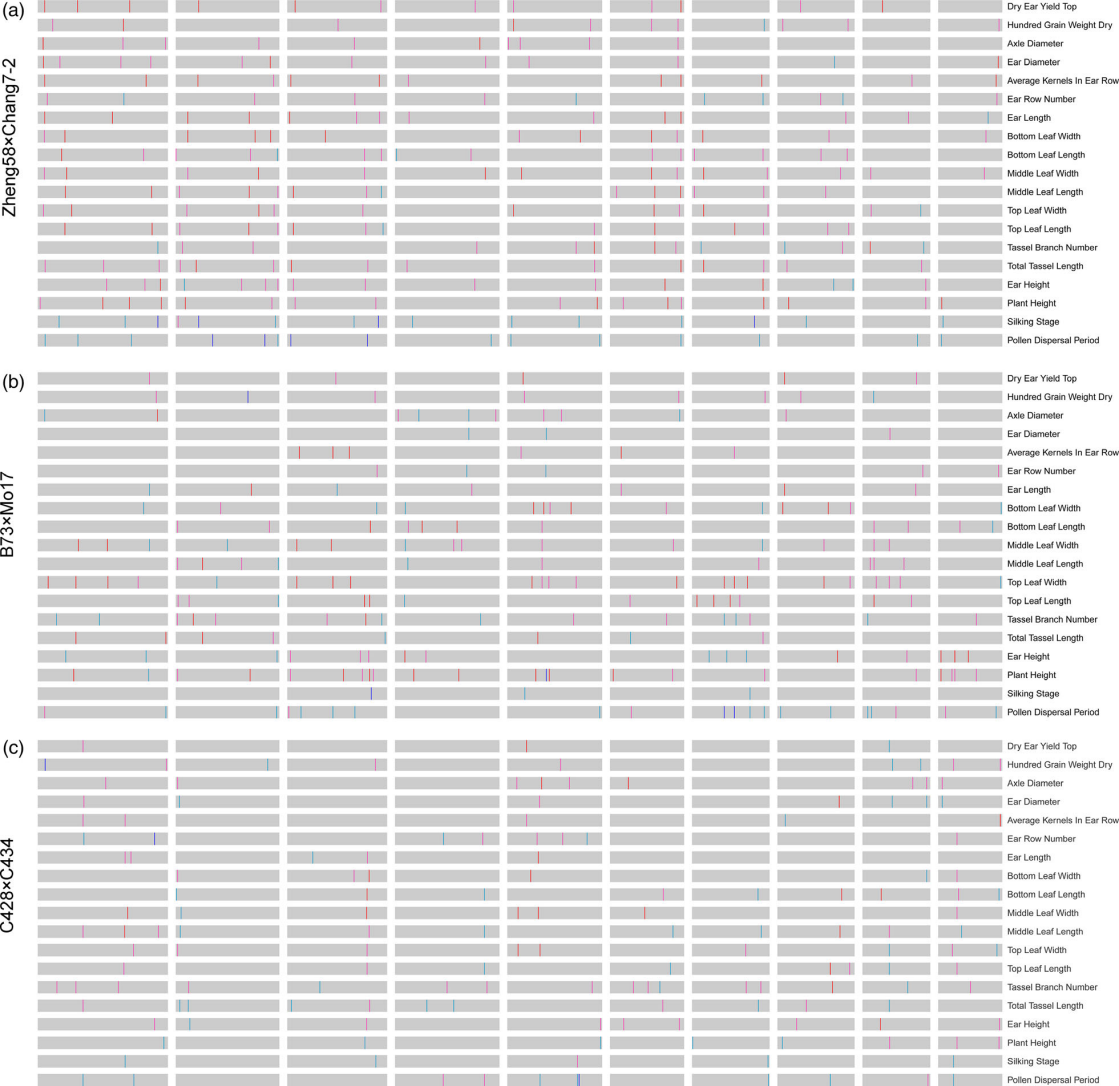
Genome‐wide QTL mapping for yield‐related traits in three F_2_ populations. (a) Plots of 256 QTLs (LOD>3.5) underlying 19 agronomic traits in Zheng58 × Chang7‐2 F_2_ population. The genomic location of each QTL peak was indicated in the maize genome by black vertical lines. (b) Plots of 214 QTLs (LOD > 3.5) underlying 19 yield‐related traits in B73 × Mo17 F_2_ population. (c) Plot of 158 QTLs (LOD > 3.5) underlying 19 yield‐related traits in C428 × C434 F_2_ population. The heterotic effects of the QTLs are indicated by deep red (*d*/*a *>* *1), light red (0 < *d*/*a *≤* *1), light blue (−1 < *d*/*a *≤* *0) and dark blue (*d*/*a *≤* *−1), respectively.

For QTLs mapping, a total of 19 agronomic traits were evaluated for F_2_ individual, inbred parents and the hybrids in Shandong Agricultural University Research Farm (eastern China, N36°01, E117°0) in 2017. The traits included flowering time, plant height, leaf length/width, kernel yield per ear and other yield component traits (Figure S2). For most traits, the F_2_ population exhibited normal distribution (Figure S3). Moreover, the parental lines were planted and phenotyped for these traits, which displayed a high amount of heterosis (Table S1), and a strong correlation of the degree of heterosis between populations could be observed (Figure S4). Furthermore, we performed genetic dissections using a composite interval‐mapping method for each population, resulting in 256, 214 and 158 QTLs identified (with LOD cut‐off >3.5) for the populations of Zheng58 × Chang7‐2, B73 × Mo17 and C428 × C434, respectively (Figure 3a‐c). As displayed in Figure 3, most traits in maize hybrids were controlled by numerous QTLs with modest or small effects, in contrast to those in rice hybrids where there were only a few large‐effect QTLs underlying one trait (Huang *et al*., 2016). Moreover, we noticed that many QTLs underlying multiple traits were located within the same genomic regions, because some traits were partially related (e.g. the QTL around the 159–169 Mb interval on chromosome 6 underling several leaf size‐related traits in Zheng58 × Chang7‐2). Additionally, there were totally 76 overlapping QTLs between populations underlying the same traits – for example the QTL on chromosome 7 for leaf length and the QTL on chromosome 5 for flowering time (Figure S5).

### Global analysis of heterotic effects

**Figure 4:**
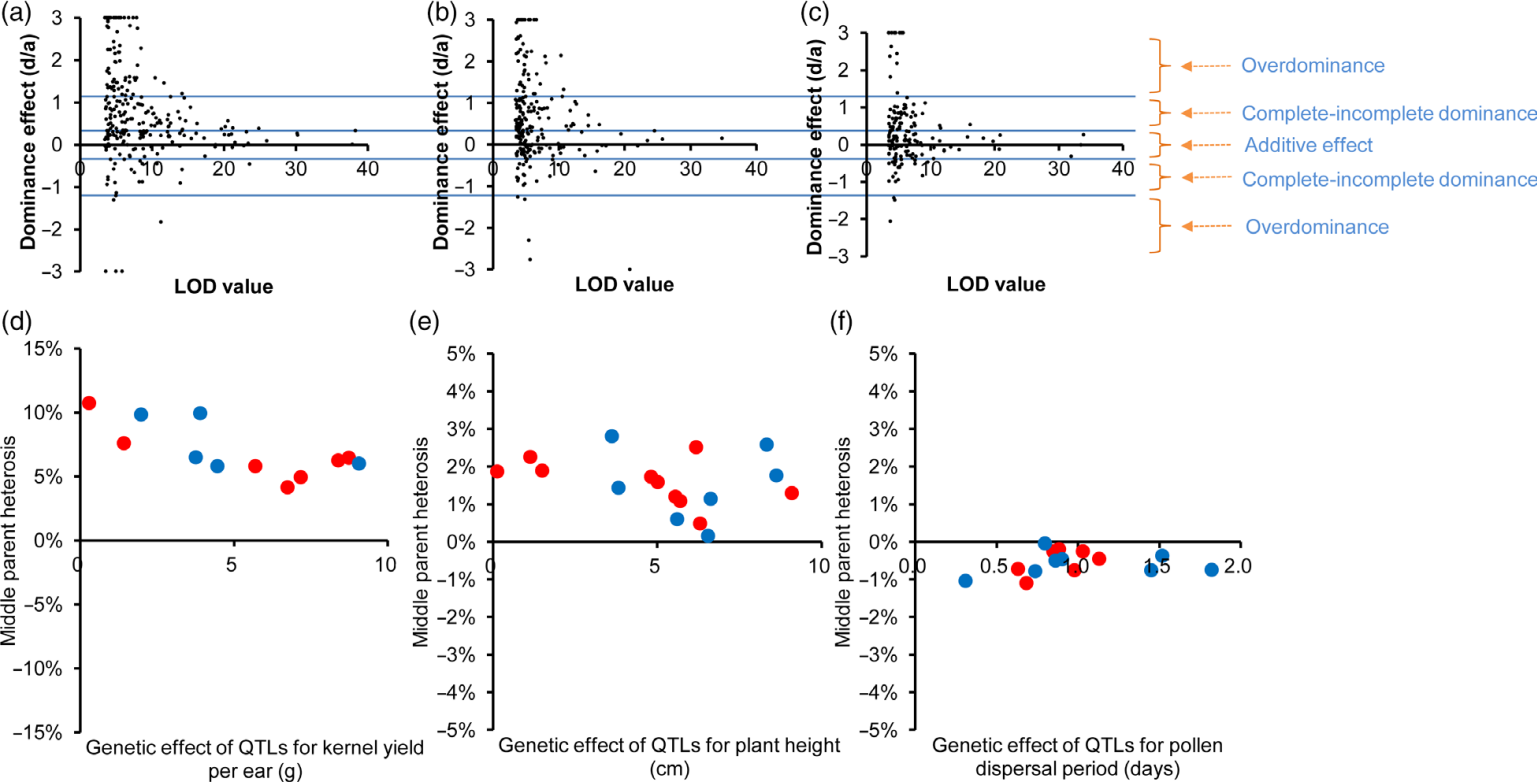
Evaluation of dominance effects for the QTLs. (a‐c) Plot of the dominance effects (*d*/*a* values) and their LOD values for the QTLs in the population Zheng58 × Chang7‐2 (a), B73 × Mo17 (b) and C428 × C434 (c). The QTLs with *d*/*a* values of >3 or <−3 are plotted to be = 3 or =−3 for display purposes. (d‐e) Plot of middle parent heterosis index and their allele effects for the QTLs underlying ear yield (d), plant height (e), and flowering time (f) in the population Zhengdan958. The alleles with better yield performance, more plant height or late flowering time that were contributed from Zheng58 and Chang7‐2 are coloured by red and blue, respectively.

We make a further inquiry that how the QTLs lead to the phenomenon of strong better‐parent heterosis in these maize hybrids. Therefore, for each QTL the genetic effect of the heterozygous genotype (MF, where M and F indicate the alleles from male and female parental lines, respectively) was compared with those of homozygous genotypes (MM and FF). When putting together the estimates of *d*/*a* for all the QTLs underlying 19 traits, the overall pattern was that – complete–incomplete dominance acted as the principle one, followed with overdominance (Figure 4a–c). It should be noticed that the number of overdominant QTLs was probably overestimated, although we have used a large population size with a high genotype resolution. One of the reasons is that there must be some pseudo‐overdominance cases (very close linkage of two advantageous alleles with repulsion phases in two inbred parents) which our genetic mapping cannot well distinguish with true overdominance cases. Another reason is that, for minor‐effect QTLs, there were more statistical errors when evaluating their phenotypic effects, leading to the false positives in overdominance cases. Hence, as expected by the hypothesis (statistical errors in minor‐effect QTLs leading to overestimated overdominance cases), we found that the number of overdominant QTLs decreases rapidly with increasing of the LOD value (Figure 4a‐c). Taken these factors into considerations, we proposed that complete–incomplete dominance should be the predominant pattern in maize heterosis, consistent with previous reports in maize (Yang *et al*., 2017a,2017b) and similar with the pattern in hybrid rice (Huang *et al*., 2016).

Considering that the population from Zheng58 × Chang7‐2 has a large sample size (*n *=* *2567), a strong yield heterosis, and an unbiased segregation distortion (25%, 50% and 25% for genotypes MM, MF and FF, respectively) across the whole maize genome (see Figure 2c), genetic dissections of yield‐related QTLs and evaluation of heterosis effects should be more powerful and precise in this population. Hence, 2567 F_2_ individuals from Zheng58 × Chang7‐2 were used for in‐depth genetic analyses. The hybrid Zhengdan958 was compared with their parental lines to evaluate the degree of heterosis. The F_1_ line showed strong heterosis for kernel yield per ear (trait‐based better‐parent heterosis BPH index = 259%), modest heterosis for plant height (trait‐based BPH index = 52%) and negative heterosis for flowering time (trait‐based BPH index = −13%). We further investigated why the level of heterosis in F_1_ (ZhengDan958) varied for different agronomic traits using the QTL data. In the F_2_ population, 15, 17 and 12 QTLs were identified for the three representative traits including flowering time, plant height and kernel yield per ear, respectively. The degree of locus‐based MPH index was strong (ranging from 4% to 10%, for each individual QTL) for QTLs underlying kernel yield, modest for QTLs underlying plant height (from 0% to 3%) and slightly negative (from −1% to 0%) for QTLs underlying flowering time (Figure 4d–f), which was consistent with the overall heterosis of each trait by comparing the F_1_ and its parental lines. This consistence indicates the level of heterosis of one trait depended on the heterotic effects of individual QTLs underlying the trait. We further addressed the question – what about the proportion of advantageous alleles from either parental line in ZhengDan958. In a previous work for hybrid rice, we had found that most of the advantageous alleles (the allele leading to more grain‐yield in rice) come from male parents (Huang *et al*., 2016). However, the situation in hybrid maize is completely different, which could be reflected from ‘the contributor sources’ (or called, ‘directions’) of QTLs for yield, height and flowering (Figure 4d–f, dots in blue and red). For total 256 QTLs identified in the Zheng58 × Chang7‐2 population, the proportions of advantageous alleles from Chang7‐2 and Zheng58 were 49.6% and 50.4%, respectively.

### Replication of heterotic effects and candidate gene analysis

**Figure 5:**
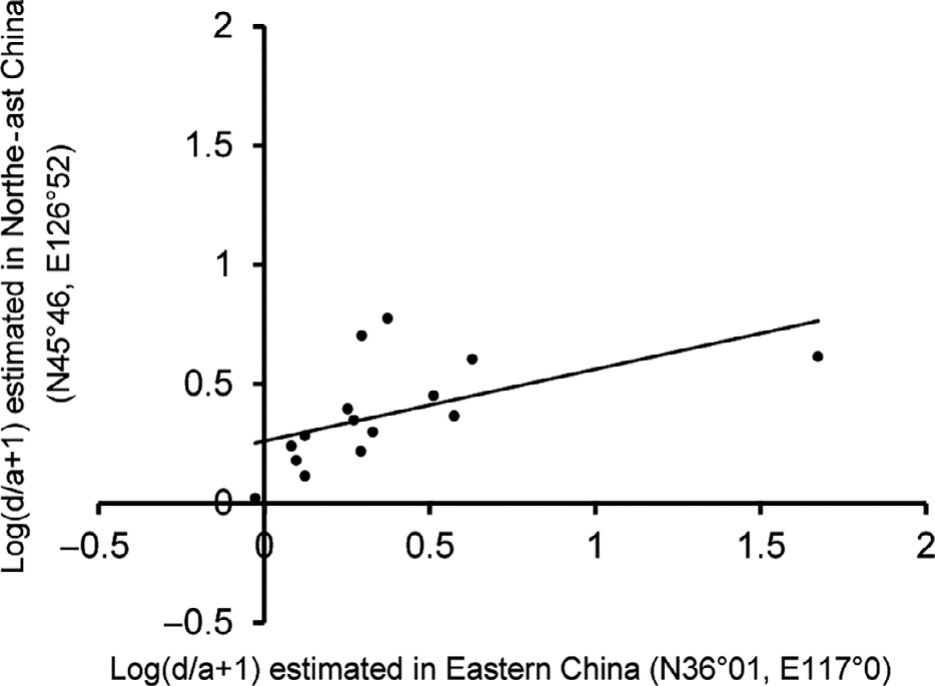
The plot of heterotic effects estimated in eastern China (N36°01, E117°0) (*x*‐axis) and north‐east China (N45°46, E126°52) (*y*‐axis) for 15 QTLs identified in maize Zhengdan958 hybrid. In the plots, the values of >log (2) represent overdominance effects, 0~log (2) represent incomplete dominance effects and <0 represent incomplete recessive effect. The Pearson correlation coefficient is indicated.

The yield‐related QTLs and their heterotic effects may be not fully stable in different environmental locations due to some environmental factors or technical noises (or called random errors). In order to evaluate the degree of these implications, we carried out an independent experiment using another set of 2225 F_2_ individuals from ZhengDan958 (Zheng58 × Chang7‐2) population which planted in Northeast Agricultural University Research Farm (N45°46, E126°52), Heilongjiang, 2018. In this replication experiment, we did not regenotyped the whole maize genomes but selected 17 major QTLs identified in Shandong (eastern China) in 2017, which underlay kernel yield per ear and yield component traits (Table 1). Molecular markers around the selected QTLs were designed, and all the F_2_ individuals in the population of Heilongjiang were genotyped at these loci. Further genetic analysis demonstrated that 15 of the 17 QTLs showed significant effects in Heilongjiang (Table 1). Moreover, the heterotic effects of the QTLs were partially consistent (Figure 5, *r* = 0.6 for the correlation of the log of *d*/*a *+* *1 values between two locations), but at several heterotic loci the *d*/*a* values had some variations between Shandong and Heilongjiang, for example from the incomplete dominance effect in Shandong to the overdominance effect in Heilongjiang, possibly due to genotype by environmental interactions.

**Table 1:** Replications of 17 heterotic loci using another ZhengDan958 F_2_ population in Heilongjiang, China

Trait	Chr.	Peak position (Mb)	Filed experiment in Shandong, China				Filed experiment in Heilongjiang, China			
			LOD	*d*/*a*	Heterotic effects	Parent with advantageous allele	*P*‐value (ANOVA)	*d*/*a*	Heterotic effects	Parent with advantageous allele
Hundred grain weight	1	36	12.4	0.21	Additive effect	Zheng58	<2e‐16	0.74	Complete–incomplete dominance	Zheng58
Kernel yield per ear	1	95	3.9	2.25	Overdominance	Chang7‐2	5.5E‐09	1.82	Overdominance	Chang7‐2
Plant height	1	218	8	2.75	Overdominance	Chang7‐2	1.9E‐07	1.32	Overdominance	Chang7‐2
Kernel yield per ear	2	55	7.4	3.26	Overdominance	Chang7‐2	7.1E‐05	3.02	Overdominance	Chang7‐2
Ear diameter	2	158	8.7	0.25	Complete–incomplete dominance	Chang7‐2	<2e‐16	0.51	Complete–incomplete dominance	Chang7‐2
Ear diameter	2	225	3.8	1.13	Complete–incomplete dominance	Chang7‐2	5.0E‐03	0.99	Complete–incomplete dominance	Chang7‐2
Kernel yield per ear	3	19	6.8	46.16*	Overdominance	Zheng58	1.1E‐05	3.11	Overdominance	Zheng58
Bottom leaf length	3	184	6.5	0.33	Complete–incomplete dominance	Zheng58	8.2E‐12	0.92	Complete–incomplete dominance	Zheng58
Kernel yield per ear	4	191	6.7	0.97	Complete–incomplete dominance	Zheng58	1.1E‐08	4.04	Overdominance	Zheng58
Bottom leaf width	5	29	6	0.96	Complete–incomplete dominance	Zheng58	1.1E‐03	0.65	Complete–incomplete dominance	Zheng58
Plant height	5	214	3.7	49.92*	Overdominance	Zheng58	1.8E‐01	−0.37	NA†	NA†
Bottom leaf length	6	101	9.5	0.79	Complete–incomplete dominance	Zheng58	1.1E‐04	1.48	Overdominance	Zheng58
Kernel yield per ear	6	169	7.4	6.36	Overdominance	Chang7‐2	6.5E‐02	5.60	NA†	NA†
Bottom leaf length	7	169	38.3	0.33	Complete–incomplete dominance	Zheng58	<2e‐16	0.30	Complete–incomplete dominance	Zheng58
Kernel yield per ear	8	55	10	0.87	Complete–incomplete dominance	Chang7‐2	4.1E‐03	1.23	Complete–incomplete dominance	Zheng58
Ear height	8	134	18.8	−0.06	Additive effect	Chang7‐2	2.0E‐04	0.05	Additive effect	Chang7‐2
Average kernels in ear row	10	138	4.2	1.36	Overdominance	Zheng58	1.0E‐06	4.95	Overdominance	Zheng58

*For the two loci, there was a great phenotypic distinction of heterozygous genotypes (MF) to homozygous genotypes (MM and FF), but very small differences between two homozygous genotypes (i.e. between MM and FF), which resulted in outrageously high *d*/*a* ratios.

^†^For the two QTLs with less significant effects (*P* > 0.05) in Heilongjiang, the heterotic effects and the parent with advantageous allele in Heilongjiang cannot be well evaluated.

**Figure 6:**
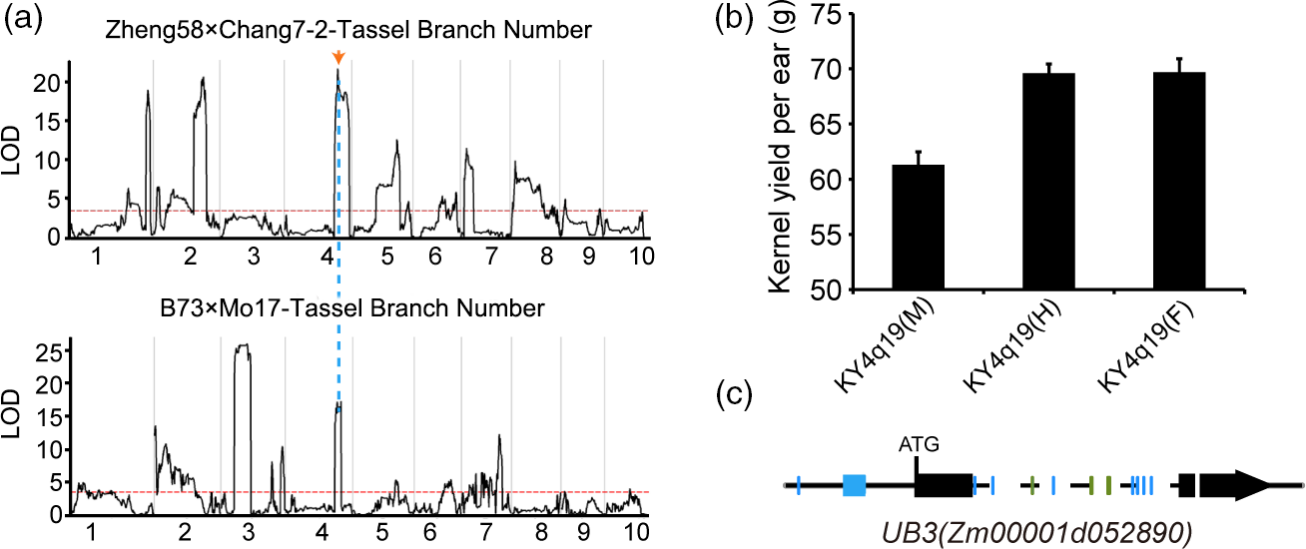
Candidate gene analysis of a major QTL KY *4q19*. (a) QTL mapping of tassel branch number in Zheng58 × Chang7‐2 and B73 × Mo17 F_2_ populations. The threshold value (LOD = 3.5) is presented by a red horizontal dashed line, and the location of the QTL peak is presented by a blue vertical dashed line. (b) The performances of kernel yield per ear for three genotypes of the *KY4q19* locus in the Zhengdan958 population in Shandong. (c) Sequence differences (coloured in blue and green for SNPs and indels, respectively) between parents Zheng58 and Chang7‐2 for the candidate gene *UB3* in the *KY4q19* locus.

We performed detailed analyses for one heterotic locus *KY4q19* which contained one candidate gene (the ortholog of *OsSPL14* in maize) and showed variations in heterotic effects between the two locations Shandong and Heilongjiang. In the hybrid rice of *indica*‐*japonica* crosses, *OsSPL14* (also known as *IPA1*) was found to be one of the key genes contributing to heterosis advantage, displaying a strong overdominance effect (Huang *et al*., 2016; Jiao *et al*., 2010; Miura *et al*., 2010). One of its orthologs in maize, *ub3* (Gene ID: *Zm00001d052890*), has been reported to be the causative gene of the QTL underlying plant architecture in maize (Chuck *et al*., 2014; Liu *et al*., 2015). This QTL (named as *KY4q19* here, containing the candidate gene *ub3*) was also identified in our populations, with large effects on regulating tassel branch number and kernel yield per ear (Figure 6a). For kernel yield per ear, the *KY4q19* heterozygote increased ~13.5% more yield advantage than the homozygous male genotype *KY4q19(M)* (Figure 6b), acting through the way of nearly complete dominance in Shandong (*d*/*a *=* *0.97). In the experiment of Heilongjiang, a strong overdominance effect was observed for the *KY4q19* heterozygote (*d*/*a *=* *4.04). Sequence comparisons between parents showed that there were no differences in coding regions of *ub3*, but many variants in the promoter regions (Figure 6c). Hence, the heterotic effect of *ub3* in yield probably resulted from an optimal expression level in its heterozygous state, which interestingly was consistent with the situation in rice (Huang *et al*., 2016; Liu *et al*., 2015).

### Discussion and implication

In order to dissect the genetic basis of heterosis, numerous studies demonstrated that the control of heterosis is complex in plants (Birchler, 2016; Huang *et al*., 2016; Kusmec *et al*., 2017; Li *et al*., 2016; Luo *et al*., 2011; Riedelsheimer *et al*., 2012; Wang *et al*., 2016; Yang *et al*., 2017a,2017b; Yao *et al*., 2013; Zhou *et al*., 2012). Due to the complex maize genome and the complex genetic architecture of heterosis traits, genome‐wide high‐resolution identification of heterotic loci and comprehensive evaluation of their genetic effects in heterosis have been difficult in maize hybrids. In this study, we constructed three high‐density genotype maps and identified 628 loci underlying 19 yield‐related traits overall 5360 F_2_ individuals, which provide a comprehensive list of the heterotic loci in maize hybrids and show that complete–incomplete dominance (the major one) and overdominance effects (the secondary one) for heterozygous genotypes exhibited an important role in heterosis. Of note, the random F_2_ populations represent a normal distribution (Figure 2c) which avoids the segregation distortion effect. The results provide a committee resource for breeders and seed industry to improve the potential yield by marker assistant selection and genomic prediction breeding (Frisch *et al*., 2010; Schrag *et al*., 2007, 2009, 2010, 2018; Windhausen *et al*., 2012).

Maize was domesticated from teosinte (*Zea mays* ssp. *parviglumis*) approximately 9000 years ago and has become the most productive and highest value commodity crop across the world. One of the main reasons for its rapid popularity in agriculture production globally is the effective use of heterosis. The large‐scale genetic and genomic analyses in maize hybrids provided a number of important heterotic loci that may be quite useful in hybrid breeding and future functional studies and showed that the strong better‐parent heterosis in maize profited from numerous advantageous alleles interspersing among parental lines. In maize hybrid, there were many QTLs with modest or small effects (probably owing to the presence of multiple paralogs and extensive variation in noncoding regulation regions), and the proportions of their advantageous alleles are much the same between male and female parents (Figure 4). The pattern is distinct from self‐fertilizing plant species (e.g. rice), in which advantageous alleles from multiple large‐effect QTLs had the chance for pyramid during long‐term breeding of inbred lines (Huang *et al*., 2016). Hence, the mating system (complete outcrossing) and the genome constitution (more paralogs and more intergenic regions) may partially explain the causes of strong heterosis in maize. Moreover, the development of high‐throughput, high‐density genotyping methods not only provide a resource to discover the genetic basis of heterosis and germplasm diversity, but also benefit to exploiting heterosis‐related SNPs and enriching rare advantageous alleles, which could help breeder to improve the hybrid agronomic performance in maize breeding populations.

Another objective of this work is to seek for the common genetic patterns and causative genes contributing to heterosis in plants. From the view of the genome‐wide pattern, many heterotic loci with incomplete dominance effects appeared to be the major cause in both rice and maize, with a few overdominance loci acting as the secondary factors. For the key gene contributors, clues have been detected for some important heterosis‐related candidates in hybrid maize or hybrid rice which were orthologous to those well‐known heterosis genes in other plant species (e.g. *OsSPL14* in rice and *ub3* in maize; *Hd3a* in rice and *SFT* in tomato), probably indicating convergent selection during hybrid breeding of a common set of core nodes related to plant architecture and flowering time pathways. Fine tuning of the function of these important genes related to plant architecture and flowering time probably lead to the occurrence of heterosis in plants.

Although both previous studies and our study showed complete–incomplete dominance could explain a large proportion of heterosis advantage, epistasis is considered as one of the important causes for heterosis in hybrid plants. The complexification of epistatic is the remaining challenge in heterotic studies. The challenge includes the following: (i) to search for potential epistasis interactions for *n* loci, *n* ^2^/2 locus–locus interactions need be tested; and (ii) for each locus–locus pairs, there are at least 9 (i.e. 3*3) allelic combinations. The current analysis algorithm for the epistasis interactions and the limited population size prevent us from conducting an effective epistasis analysis. With the development of well‐designed genetic populations and efficient methods for epistasis analysis, we believe more genetic findings on epistasis interactions will help a better understanding of heterosis in maize and other corps.

## Experimental procedures

### Whole‐genome sequencing

Three hybrid combinations in maize (Zheng58 × Chang7‐2, B73 × Mo17 and C428 × C434) were selected to construct F_2_ populations, generating totally 5360 lines for whole‐genome sequencing and phenotyping. For each F_2_ individual in the hybrid maize populations, the genomic DNA was extracted from fresh leaf tissues using N96 DNAsecure Plant Kit (TianGen). Sequencing libraries were constructed using TruePrep Tagment Enzyme (Tn5 transposase) with an insert size of 300‐500 bp (Picelli *et al*., 2014). DNA samples of each F_2_ individual were bar‐coded in the sequencing libraries and amplified using TruePrep Amplify Enzyme. The indexed DNA samples of ~384 F_2_ individuals with different barcodes were mixed together with a nearly equal molar concentration. Each mixture was loaded into one lane of the Illumina HiSeq4000 system.

### Genotype calling of the populations

Overall, six parental lines (Chang7‐2, Zheng58, Mo17, B73, C434 and C428) and 5360 F_2_ individuals in the three populations were sequenced, generating 150 bp paired‐end reads containing totally ~2.4 terabase sequences. Each parental line had approximately 30× genome coverage, and each F_2_ individual in the hybrid maize populations had approximately 0.2× genome coverage. The sequence reads of the six parental lines were aligned against the maize reference genome (AGPv4, downloaded from the Gramene database, ftp://ftp.gramene.org/pub/gramene/release53/data/fasta/zea_mays/dna/) with the BWA package (version 0.7.1) using default parameters, and PCR duplicates were removed by the ‘MarkDuplicates’ module in the Picard tools (version 1.119) (Li and Durbin, 2009). The raw reads were also re‐aligned for the highly polymorphic regions using the ‘IndelRealigner’ function in the software package GenomeAnalysisTK (version 3.4.0; DePristo *et al*., 2011). The sequence variants between parental lines were called using ‘UnifiedGenotyper’ in the GenomeAnalysisTK. The paired‐end reads of each F_2_ individual were mapped onto the maize reference genome sequence using the software package SMALT (version 0.5.7). Only the uniquely mapped reads were used for subsequent SNP calling. Genotype calling of each F_2_ individual was carried out based on the SNP alleles between parents, using the pipeline SEG‐Map (Huang *et al*., 2009). For each F_2_ population, the genotypic data of each F_2_ individual were combined together for bin map constructions.

### Construction of neighbour‐joining tree

Resequencing reads from 281 published maize inbred lines (102 teosintes, landraces and modern inbred lines from maize HapmapII and 179 temperate maize breeding lines from maize HapmapIII project) were incorporated with data of six parental lines in this study, and SNPs were called by using GATK pipeline. IBS distance matrix of 287 lines was calculated by Plink (v1.07) with parameters –maf 0.05 –map3 –noweb –cluster –distance matrix –out. Then, the neighbour‐joining tree was constructed by phylip (v3.696) with default parameters.

### Planting and phenotyping of the F_2_ individuals

The six inbred lines were used for population construction. In brief, sixty kernels from a single ear of Chang7‐2, Zheng58, Mo17, B73, C434 and C428 were planted in Shandong Agricultural University Research Farm (eastern China, N36°01, E117°0), Tai'an, Shandong province, China, in 2016. The F_1_ seeds from crosses of Zheng58 × Chang7‐2, B73 × Mo17 and C428 × C434 were generated in this season. Subsequently, the F_1_ seeds of three combinations were planted for selfing in Shandong Agricultural University Research Nursery (South China, N18°24, E109°01), Sanya, Hainan province, China, in 2016 winter. For each combination, the F_2_ population were planted in Shandong Agricultural University Research Farm (eastern China, N36°01, E117°0), Tai'an, Shandong province, China, in 2017. All the F_2_ individuals were grown in the consecutive farmland with well‐distributed soil status in the field. The ears of all the F_2_ individuals were open pollinated for evaluating yield traits. Detail description of phenotyping works for 19 agronomy traits can be found in Figure S2.

### Quantitative genetic analyses

In each F_2_ population, QTL analysis of 19 agronomic traits was carried out using the composite interval‐mapping (CIM) method with QTL Cartographer (version 2.5) with a step size of 2 cM, the cross type of SF_2_ and a window size of 10 cM. The calculations of LOD values were based on likelihood ratio tests allowing both additive and dominance effects (Silva Lda *et al*., 2012). QTLs with LOD value higher than 3.5 were used for follow‐up heterosis analyses. After the QTLs were identified, genetic effects were assessed through comparisons of both homozygous genotypes and the heterozygous genotypes to search for advantageous alleles. The average phenotypic measurements of heterozygous genotypes and homozygous genotypes were further calculated for the estimation of the dominance effect index (*d*/*a*) and the MPH index for each QTL. According to the estimates of dominance effects (short for *d*) with those of additive effects (short for *a*), each yield QTL underlying a certain trait could be divided into one of the five cases: overdominance (*d*/*a* > 1.25, in which the MF genotype displays more yield than both MM and FF), complete–incomplete dominance (0.25 < *d*/*a *≤* *1.25), additive effect (−0.25 < *d*/*a *≤* *0.25), incomplete recessive (−1.25 < *d*/*a *≤* *−0.25) and underdominance (*d*/*a *≤* *−1.25, in which the MF genotype displays less yield than both MM and FF). In the replication experiment, another set of 2225 F_2_ individuals from ZhengDan958 population were planted in Northeast Agricultural University Research Farm (N45°46, E126°52), Heilongjiang, in 2018 for phenotyping. The F_2_ individuals were all genotyped at 17 target loci using a ligase detection reaction method. Statistic tests were performed through the analysis of variance (ANOVA), and the heterotic effects (the estimates of *d*/*a* values) were calculated as that in Shandong.

### Candidate gene analysis

The protein sequences of *ub3* were compared with its homologs in maize and rice using BLAST, ClustalW and MEGA5. PCR primers were designed for *ub3* for Sanger‐based sequencing of the parental lines in AB3730. The PCR primers include *ub3 *+* *1106R: 5′CTGAGGGCCAACTGATCATGCCA3′, *ub3 *+* *2211F: 5′CTGGCCTAGCCTAGTAGGAAGAGA3′, *ub3 *+* *2394R: 5′GGTCAAAGCCAGTGGGAATGCT3′, *ub3 *+* *3782R: 5′CTGGATGCTCTAGCCTCTAGGCA3′, *ub3*‐114F: 5′GTCAGCCAGCCAGCCCAAGA3′, *ub3 *+* *1001F: 5′GTGCTCATTGCTTAGTCTGCCTCA3′, *ub3 *+* *69R: 5′GCCGGCGTCCTCGAAGTAGAT3′, *ub3*‐3046F: 5′GGCGTGTTTAGCATTAGCTCC3′, *ub3*p2‐3F: 5′CACTAGAACCAAACGGTATACATGAC3′, *ub3*p2‐3R: 5′ATACCTGAGCCGACGATCTCAT3′, *ub3*p3‐3F: 5′TTGTAGACGCCAGGTACTTGCGCTTGT3′, *ub3*p3‐3R: 5′GTCATGTATACCGTTTGGTTCTAGTG3′.

The first eight primers were designed in this study, and the last four primers (*ub3*p2‐3F/R and *ub3*p3‐3F/R) were according to a previous study (Liu *et al*., 2015). For exon and intron regions, the *ub3*‐114F/*ub3 *+* *3782R pair was used for long fragment amplifications, while *ub3*‐114F, *ub3 *+* *1106R, *ub3 *+* *1001F, *ub3 *+* *2394R, *ub3 *+* *2211F and *ub3 *+* *3782R primers were used for sequencing. For promoter regions, the *ub3*‐3046F/*ub3 *+* *69R pair was used for long fragment amplifications, while the remaining primers (*ub3*‐3046F, *ub3 *+* *69R, *ub3*p2‐3F/R and *ub3*p3‐3F/R) were used for sequencing.

## Accession numbers

The DNA sequencing data of the six parental lines are deposited in the European Nucleotide Archive under accession numbers PRJEB30082 (http://www.ebi.ac.uk/ena/data/view/PRJEB30082).

## Competing interests

The authors declare no competing financial interests.

## Author contributions

H.L., X.H. and X.S.Z. designed the research; Y.D., L.Z., X.L., C.K., M.S., F.L., Y.W., Y.Z., B.L. and X.Y.Z. contributed to create the genetic population and phenotyping for the whole related traits. Q.W., X.Y., J.L., M.C., C.Z., Q.T., Y.L., D.F., J.S. and Q.F. performed the genome sequencing and expression analysis. X.H., H.L., J.Y. and M.C. performed the data analysis and genetics analysis. X.H., H.L., B.H., J.Y., J.L. and X.S.Z. discussed the data and wrote the paper.

## Supporting information


**Figure S1** Genome‐wide distribution of the genetic variation throughout the maize genome for Zheng58 × Chang7‐2, B73 × Mo17 and C428 × C434, respectively.

**Figure S2** Detail descriptions of phenotyping works for 19 agronomical traits.

**Figure S3** Distribution of each phenotype across Zhengdan958 F_2_ populations, parental lines, and F_1_ hybrid lines.

**Figure S4** The plots and the correlation coefficient of MPH and BPH of the 19 traits between F_2_ populations.

**Figure S5** Overlapping QTLs between populations.

**Table S1** Phenotype data of parental inbreds and F1 hybrids for all 19 traits in three hybrids.
