# Supplementary figures and images for: Genome‐wide identification and analysis of heterotic loci in three maize hybrids
Source: Plant Biotechnol J. 2019 Jun 27;18(1):185–94. doi: 10.1111/pbi.13186 (PMC6920156; doi:10.1111/pbi.13186)

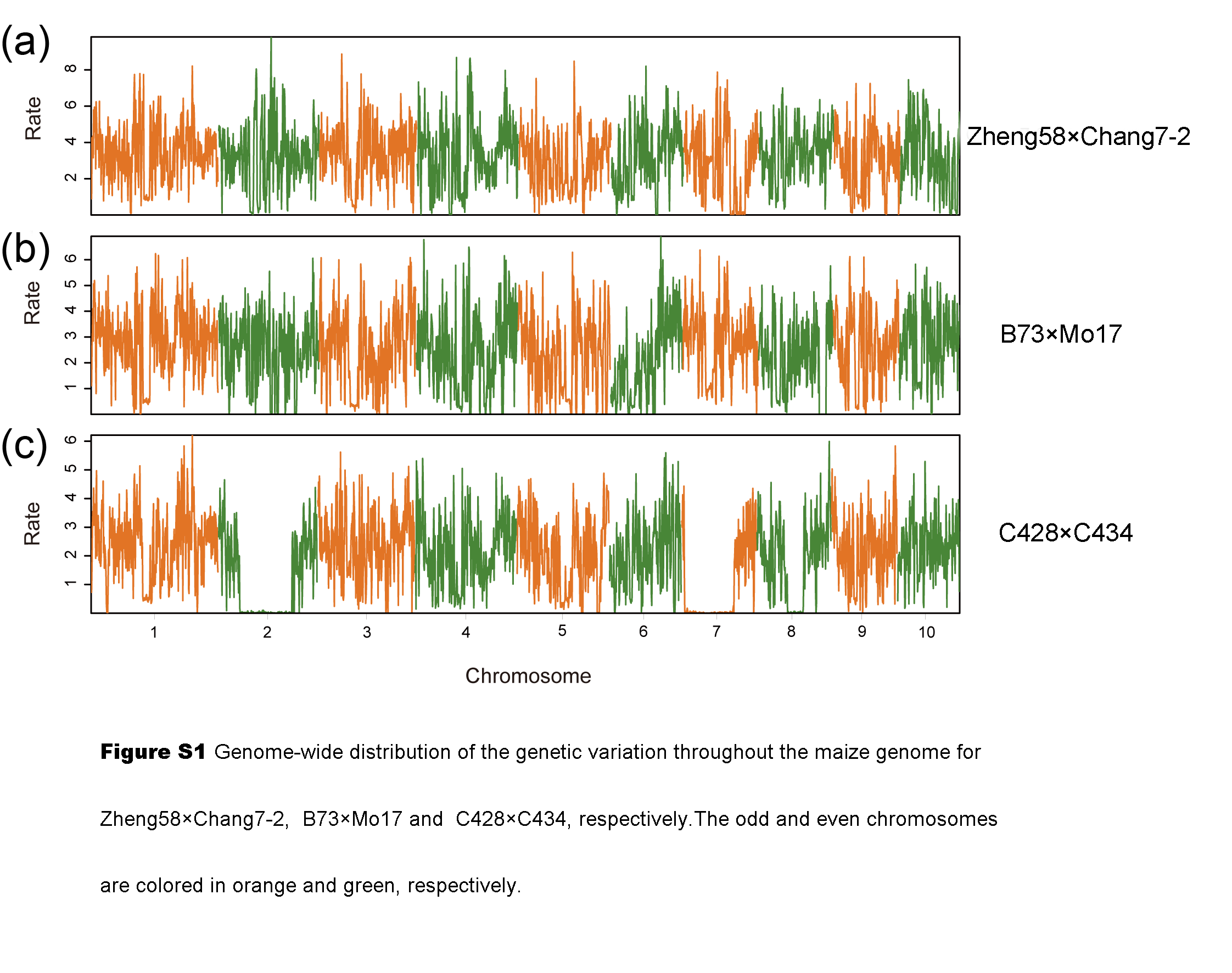

Supplement: Supplementary file 1 — Figure S1 Genome‐wide distribution of the genetic variation throughout the maize genome for Zheng58 × Chang7‐2, B73 × Mo17 and C428 × C434, respectively. [file PBI-18-185-s005.tif]

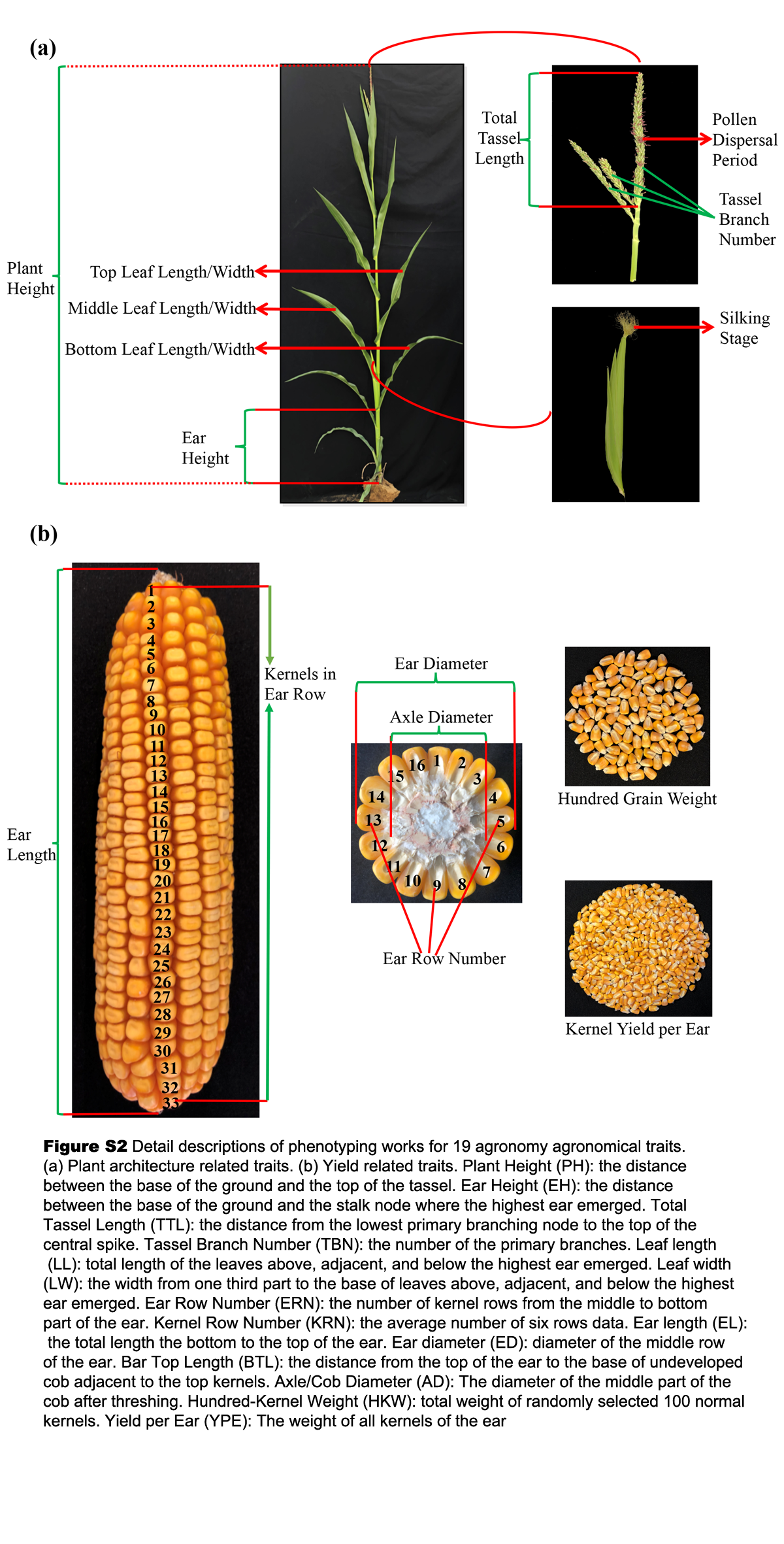

Supplement: Supplementary file 2 — Figure S2 Detail descriptions of phenotyping works for 19 agronomical traits. [file PBI-18-185-s004.tif]

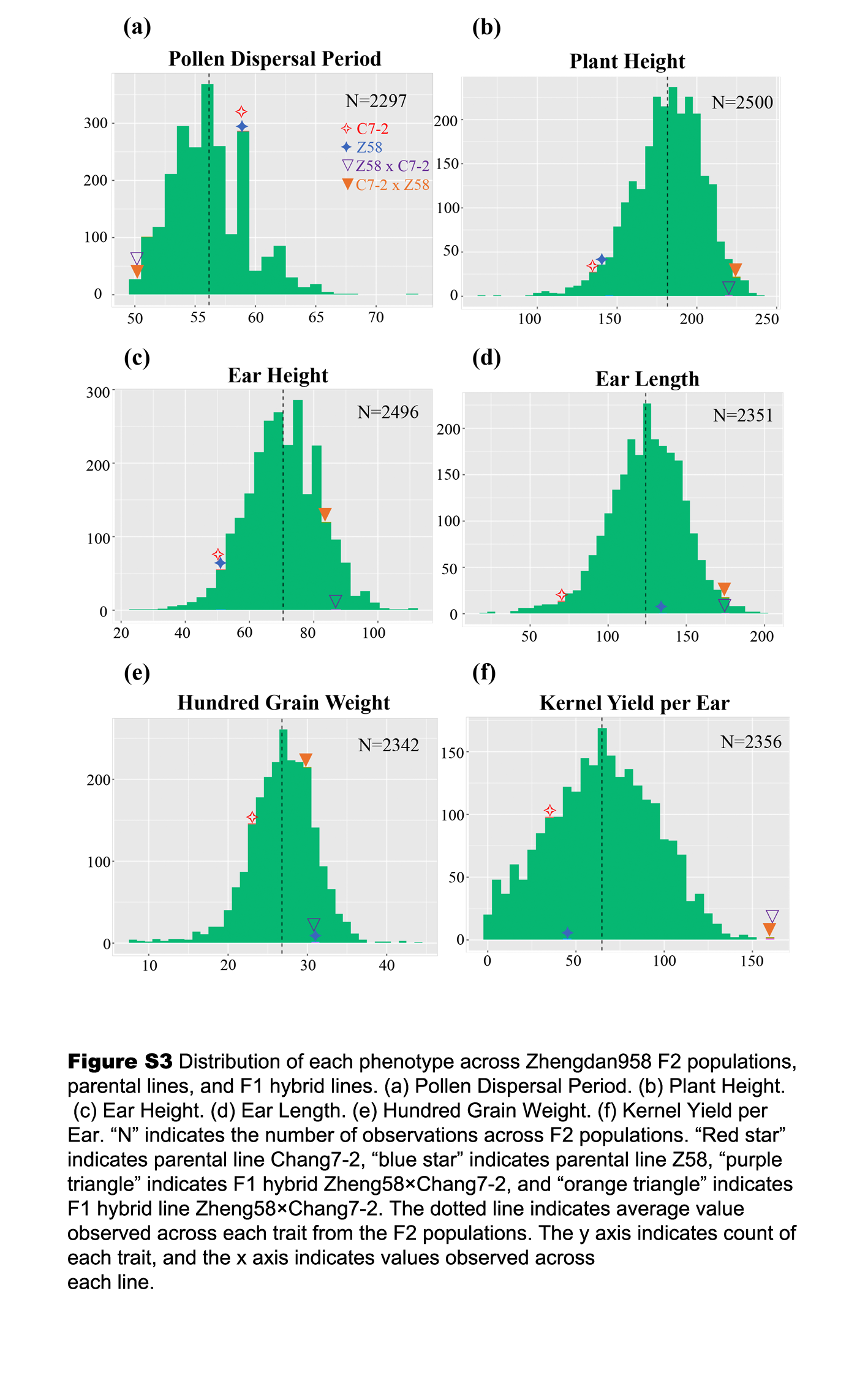

Supplement: Supplementary file 3 — Figure S3 Distribution of each phenotype across Zhengdan958 F2 populations, parental lines, and F1 hybrid lines. [file PBI-18-185-s003.tif]

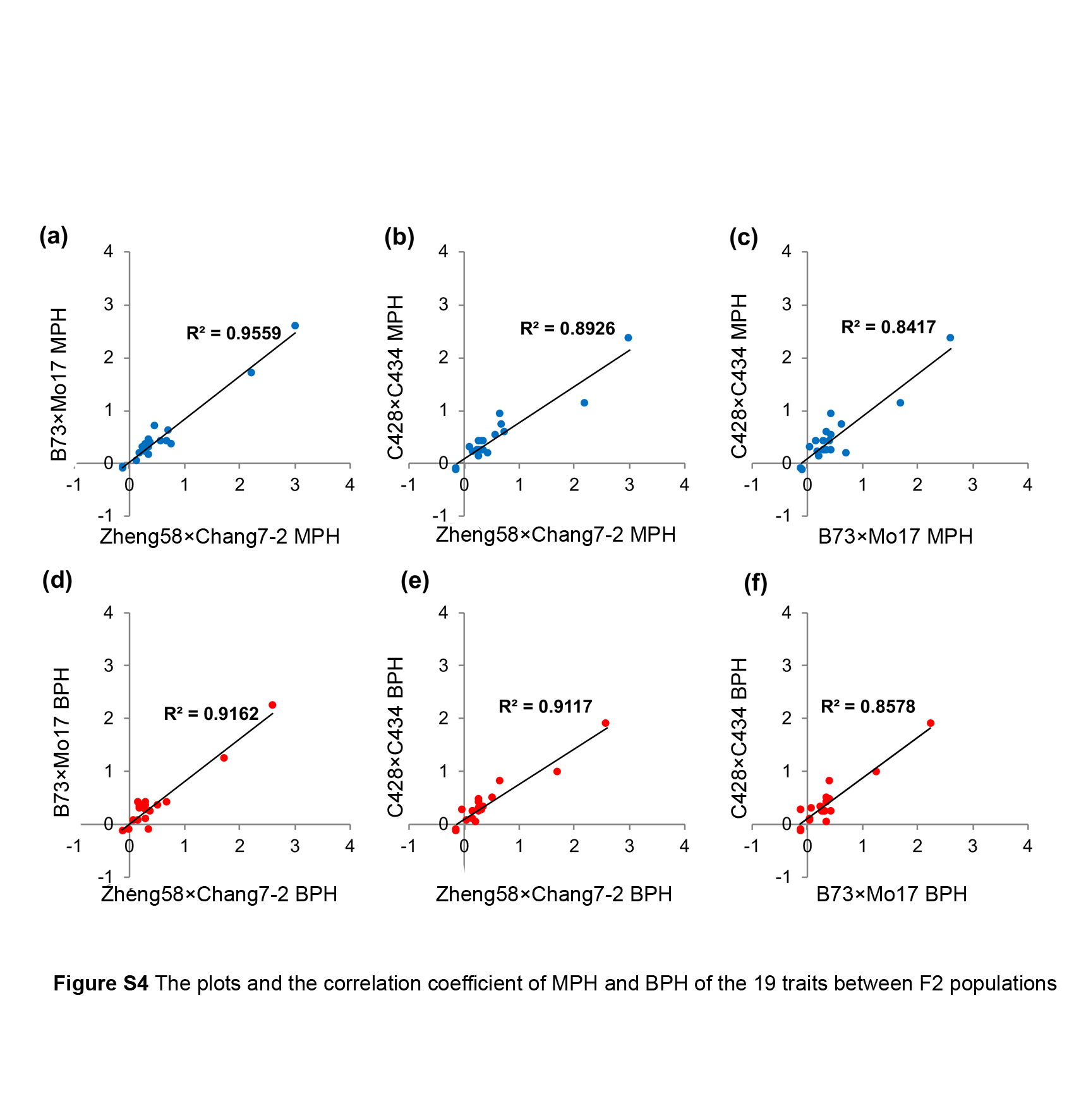

Supplement: Supplementary file 4 — Figure S4 The plots and the correlation coefficient of MPH and BPH of the 19 traits between F2 populations. [file PBI-18-185-s006.tif]

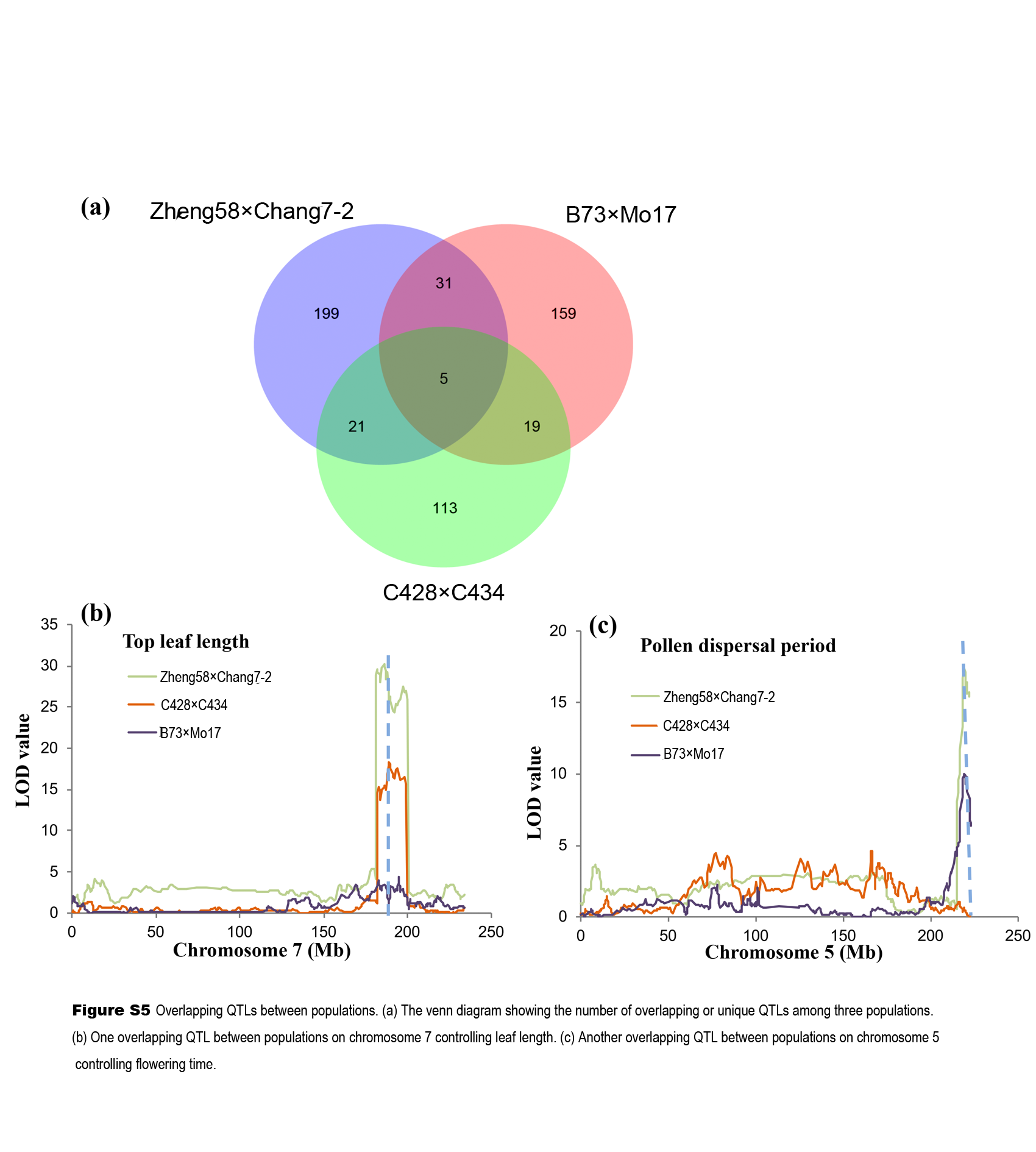

Supplement: Supplementary file 5 — Figure S5 Overlapping QTLs between populations. [file PBI-18-185-s002.tif]
