# Supplementary material for: Genome‐wide identification and analysis of heterotic loci in three maize hybrids
Source: Plant Biotechnol J. 2019 Jun 27;18(1):185–94. doi: 10.1111/pbi.13186 (PMC6920156; doi:10.1111/pbi.13186)
Supplement: Supplementary file 6 — Table S1 Phenotype data of parental inbreds and F1 hybrids for all 19 traits in three hybrids. [file PBI-18-185-s001.docx]

| Supplementary Table1 Phenotype data of parental inbreds and F_1_ hybrids for all 19 traits in three hybrids | | | | | | | | | | | |
| --- | --- | --- | --- | --- | --- | --- | --- | --- | --- | --- | --- |
| Plant height (cM) | Zheng58 | Chang7-2 | Chang7-2×Zheng58^*^ | Zheng58×Chang7-2 | Mo17 | B73 | Mo17×B73^*^ | B73xMo17 | C434 | C428 | C434×C428^*^ |
| Silking stage (Day) | 146.79 | 136.29 | 223.13 | 222.42 | 199.28 | 180.79 | 269.38 | 244.22 | 185.76 | 178.56 | 278.32 |
| Pollen dispersal period (Day) | 58.60 | 58.60 | 51.00 | 51.33 | 63.00 | 57.00 | 55.00 | 56.50 | 61.80 | 63.00 | 54.40 |
| Ear height (cM) | 58.80 | 56.20 | 51.00 | 51.00 | 58.40 | 61.80 | 53.67 | 54.75 | 59.60 | 60.00 | 53.40 |
| Total tassel length (cM) | 51.07 | 50.08 | 85.07 | 87.31 | 69.78 | 69.32 | 98.54 | 97.11 | 55.20 | 61.95 | 112.63 |
| Tassel branch number | 20.51 | 14.21 | 25.23 | 23.48 | 23.54 | 13.48 | 31.65 | 30.79 | 21.97 | 16.56 | 22.96 |
| Top leaf length (cM) | 5.77 | 12.31 | 15.94 | 11.89 | 5.67 | 5.52 | 7.58 | 7.06 | 7.77 | 9.80 | 13.86 |
| Top leaf width (cM) | 73.70 | 55.09 | 85.88 | 87.27 | 63.81 | 65.67 | 92.99 | 89.88 | 68.99 | 69.51 | 86.24 |
| Middle leaf length (cM) | 6.61 | 6.59 | 9.09 | 9.07 | 7.67 | 5.81 | 9.43 | 8.83 | 7.67 | 6.54 | 10.14 |
| Middle leaf width (cM) | 79.13 | 63.47 | 92.58 | 91.66 | 67.64 | 69.97 | 93.16 | 91.09 | 75.20 | 75.37 | 93.25 |
| Bottom leaf length (cM) | 7.00 | 7.07 | 9.42 | 9.39 | 10.31 | 5.45 | 9.08 | 8.71 | 8.10 | 6.55 | 10.30 |
| Bottom leaf width (cM) | 83.07 | 72.70 | 97.03 | 94.61 | 70.65 | 71.28 | 92.53 | 91.50 | 79.22 | 78.77 | 98.22 |
| Ear length (mm) | 7.14 | 7.23 | 9.26 | 9.42 | 7.71 | 5.11 | 8.30 | 8.29 | 7.88 | 6.40 | 10.06 |
| Ear diameter (mm) | 135.14 | 68.34 | 172.69 | 176.44 | 141.61 | 105.14 | 199.07 | 181.00 | 118.49 | 83.19 | 173.73 |
| Ear row number | 34.63 | 38.41 | 49.46 | 48.88 | 31.46 | 34.08 | 43.01 | 42.67 | 40.76 | 39.34 | 50.29 |
| Average kernels in ear row | 9.56 | 14.00 | 15.11 | 14.78 | 9.66 | 12.93 | 13.67 | 13.56 | 13.89 | 12.50 | 14.74 |
| Axle diameter (mm) | 9.56 | 14.00 | 37.92 | 38.01 | 18.90 | 12.59 | 42.49 | 33.21 | 18.92 | 16.08 | 37.43 |
| Hundred grain weight (g) | 22.26 | 23.13 | 26.95 | 26.47 | 17.83 | 22.47 | 23.67 | 23.02 | 25.36 | 20.64 | 27.89 |
| Kernel yield per ear (g) | 30.85 | 22.56 | 30.11 | 30.76 | 26.11 | 18.03 | 23.18 | 27.55 | 24.39 | 22.40 | 30.57 |
| Plant height (cM) | 43.89 | 34.94 | 157.71 | 158.50 | 39.49 | 31.29 | 127.44 | 107.12 | 57.66 | 41.30 | 166.95 |
